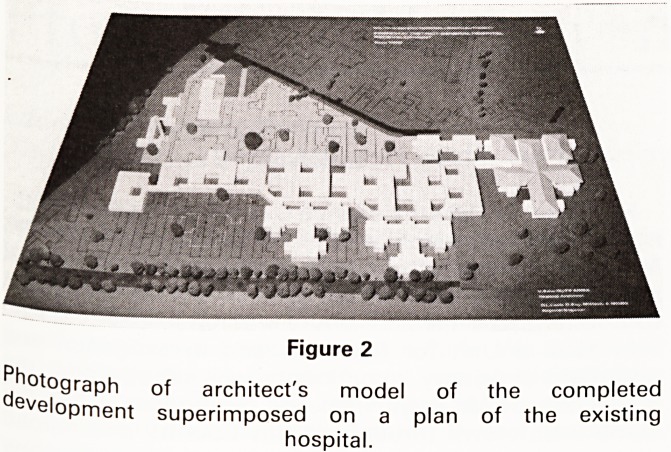# Redevelopment of Frenchay Hospital

**Published:** 1987-02

**Authors:** John S M Zorab

**Affiliations:** Consultant Anaesthetist, Frenchay Hospital


### The redevelopment of Frenchay Hospital

**Dr John S M Zorab, FFARCS,**
Consultant Anaesthetist, Frenchay Hospital

The present discussions aimed at the rebuilding of Frenchay Hospital began in 1977 under the auspices of the Avon Area Health Authority. The initial Steering Group was formalised in 1982 as a Regional Health Authority Project Team of which the author was the Consultant Member.

The brief was to plan for a replacement of the whole hospital and asked for planning to be divided into £2 million aliquots but this rapidly proved an absurd constraint and was deleted. A more fundamental requirement was for the development to be divided into a number of phases and that, at the end of each phase, the old and new parts of the hospital should function as a whole in case a subsequent phase should be delayed.

All plans, of course, had to be designed within the DHSS cost limits and were subject to county planning approval.

### SITING

The Project Team first considered a number of rebuilding options. It was eventually decided to make a start on a green grass site at the eastern end of the existing hospital and at the 'top end' of the main hospital corridor. This reduced decanting problems and allowed the remaining phases to be planned on a 'pull down and rebuild' basis. It also allowed for the preservation of some existing good quality buildings. The only 'clinical' building planned for preservation is the day hospital. The chapel, the postgraduate centre, the school of nursing, the medical records building and the Clark Hall residences are also scheduled for preservation together with the Sister's House (now, District Headquarters) and Ward 29. The latter is part of the original Frenchay TB sanatorium (opened in 1931) and is well sited for conversion to a speech therapy Unit.

### OVERALL CONCEPT AND LAY-OUT

The overall concept is for a two-storey hospital built either side of a main corridor with ward blocks mainly on the south side and clinical departments on the north side. The hospital will ultimately function around a core comprising the accident and emergency department, the outpatient department, the diagnostic imaging department, the pathology department and the two groups of operating theatres. These will form a high technology core catering for the diagnostic and therapeutic needs of both outpatients and in-patients. This core will lend itself to the changing emphasis from in-patient to outpatient care and provide sufficient flexibility to meet the relentless advance of high technology medicine.

The wards are planned in groups adjacent to the facilities on which they will mainly depend. Thus the surgical wards will be closest to the theatre complexes with the medical wards more remote but provided with on-site facilities to meet as many of the diagnostic and therapeutic needs as possible.

One of the constraints under which planning has to take place is that of providing a *replacement* hospital and not creating a host of new developments. Some development is, of course, essential but every request has to be assessed and scrutinised with a view to containing both capital and revenue implications within the existing budget.

**Figure 1**

Plan view of the completed development. Numbers indicate proposed phasing. Letters indicate various areas.

1. Ten ward block and burns unit
2a. Accident Centre with 7 theatre suite over
2b. 6 ward block
2c. Two two-ward blocks
3. Rehabilitation and neuropathology
4. Diagnostic imaging
5a. Main entrance and outpatients with 5 theatre suite over
5b. 6 ward block
6. General pathology and CSSD
7. Catering

A. Medical illustration
B. Ward 29 (Speech therapy)
C. Burns unit entrance
D. Accident and Emergency entrance
E. Development area
F. Main entrance
G. Boiler house
H. Kitchens
I. Development area
J. Conservation area
K. Chapel
L. Day Hospital
M. Garden area
N. District Headquarters

### CONSEQUENCES OF SITING

For the last few years, a number of preliminary but related works have been going on such as the installation of a new drainage system to the whole site, a new telephone system, a new medical gas installation, a new boiler house (nearing completion) and new accommodation for the department of medical illustration and the pain clinic whose premises have to be removed to make way for Phase I.

A number of other problems have also had to be resolved. Frenchay Hospital is very conscious of its fine

**Figure 2**

Photograph of architect's model of the completed development superimposed on a plan of the existing hospital.

selection of trees and although a number of trees have inevitably been marked for felling, substantial plantations have been planted in the hospital grounds to allow for rapid replacement of trees as the development progresses.

Since the Phase I site spreads over the existing hospital ring road, one of the earliest tasks was to re-route the road to provide access to the building site. A residential block had to be demolished and a number of car parking areas were lost.

### PHASE I

Phase I started on July 7th, 1986 and comprises a ten-ward block together with an integral burns unit, consultant and other 'departmental' accommodation for the specialties that will be housed in the new wards and a connecting corridor to the existing hospital. Frenchay will also see the installation of its first lift!

The ten wards are divided into a trefoil ward block with three wards on each floor and two single blocks with one ward on each floor. The burns unit lies between the single blocks on the ground floor. It has its own ambulance access and is easily accessible from the hospital's helicopter landing site. The adjacent wards house the adult plastic surgical patients and the oral surgical patients. In the trefoil block, the wards for the elderly are on the ground floor and will have access directly on to a landscaped garden area with the Day Centre adjacent to the garden. Following the completion of Phases II and III, the new children's wards and hospital school will also have access to this garden area.

The remaining wards in Phase I will house the general medical patients and the orthopaedic patients. The general medical wards will include facilities such as a coronary care unit, a pacing room, a stoma clinic, an endoscopy room and possibly some imaging equipment. The conjunction of the orthopaedic wards to those for the care of the elderly is seen as being an important relationship.

### SUBSEQUENT PHASES

It had been hoped that the necessary capital would become available for each successive phase to follow on with the least possible delay. Current projections, however, are that no further redevelopment capital can be found before the next planning decade which starts in 1994 unless there is a marked change in government policy.

Nevertheless, there is an urgent need for the next major phase which will comprise the accident and emergency department, the first of the two operating theatre suites (7 theatres), part of the new diagnostic imaging department, the intensive therapy unit and, possibly, additional ward blocks.

One of the effects of the delay is that substantial capital will have to be spent on the existing structure in order to keep the service going until the redevelopment can continue. Even if capital becomes available, the entire redevelopment is likely to take five, six or even seven phases and, since each phase is likely to take three years from start on site to completion of commissioning, a very extended building programme of some twenty years is probably inevitable.

This could undoubtedly be shortened if the number of phases were reduced, each phase being enlarged, but there is a limit to which this can be achieved owing to the need to pull down the existing hospital in order to provide the available site for the new. Nevertheless, the overall plans for the final development suggest that a well-designed, flexible and reasonably attractive building will result which will accommodate the best possible functional relationships as well as providing for a continuing swing from in-patient to outpatient care.

This will not come in the working life-time of most of the present consultant staff but those newly appointed will, it is hoped, have the opportunity to treat their patients in surroundings appropriate to the beginning of the next century.

## Figures and Tables

**Figure 1 f1:**
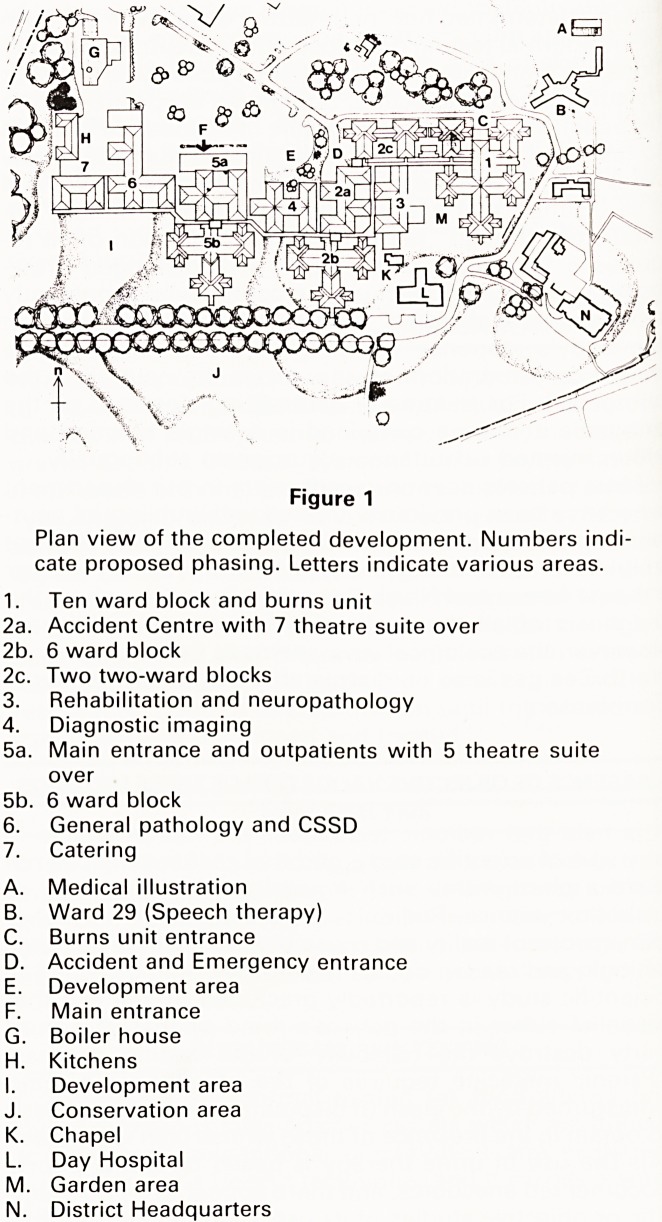


**Figure 2 f2:**